# Mitochondrial function declines with age within individuals but is not linked to the pattern of growth or mortality risk in zebra finch

**DOI:** 10.1111/acel.13822

**Published:** 2023-03-20

**Authors:** Pablo Salmón, Neal J. Dawson, Caroline Millet, Colin Selman, Pat Monaghan

**Affiliations:** ^1^ School of Biodiversity, One Health and Veterinary Medicine University of Glasgow Glasgow UK; ^2^ Institute of Avian Research “Vogelwarte Helgoland” Wilhelmshaven Germany

**Keywords:** ageing, avian models, mitochondria, red blood cells, senescence, survival

## Abstract

Mitochondrial dysfunction is considered a highly conserved hallmark of ageing. However, most of the studies in both model and non‐model organisms are cross‐sectional in design; therefore, little is known, at the individual level, on how mitochondrial function changes with age, its link to early developmental conditions or its relationship with survival. Here we manipulated the postnatal growth in zebra finches (*Taeniopygia guttata*) via dietary modification that induced accelerated growth without changing adult body size. In the same individuals, we examined blood cells mitochondrial functioning (mainly erythrocytes) when they were young (ca. 36 weeks) and again in mid‐aged (ca. 91 weeks) adulthood. Mitochondrial function was strongly influenced by age but not by postnatal growth conditions. Across all groups, within individual *ROUTINE* respiration, *OXPHOS* and *OXPHOS* coupling efficiency significantly declined with age, while *LEAK* respiration increased. However, we found no link between mitochondrial function and the probability of survival into relatively old age (ca. 4 years). Our results suggest that the association between accelerated growth and reduced longevity, evident in this as in other species, is not attributable to age‐related changes in any of the measured mitochondrial function traits.

## Graphical abstract

We show that, within individuals, age‐related changes in blood cell mitochondria function are evident by mid‐adulthood (91 weeks) in captive zebra finches. Routine cellular respiration and respiration linked to ATP synthesis (*OXPHOS*) declined with age, while mitochondrial proton *LEAK* increased. Mitochondrial function did not relate to experimental manipulations of postnatal growth rate or to the probability of survival into old age (ca. 4 years).

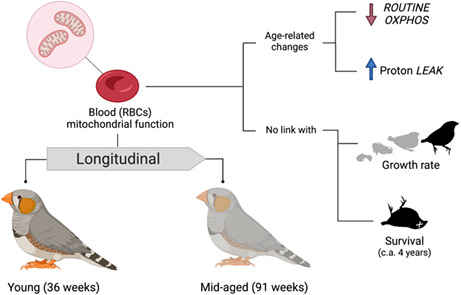

**Abbreviations**
CS: citrate synthase activity
OxCE: apparent OXPHOS coupling efficiency
OXPHOS: oxidative phosphorylation
RBC: red blood cell

Comparisons of different age groups of humans and other animals often report decreased mitochondrial functionality with age (e.g., Hebert et al., 2010). However, due to the terminal sampling approaches used in many studies, the observed changes with age may be attributable to differential survival of phenotypes rather than due to within individual changes in mitochondrial function. Birds possess functional mitochondria within their red blood cells (RBC), enabling repeated measures within the same individual over time in a minimally invasive way (Dawson & Salmón, 2020; Stier et al., 2013). Accelerated growth rate in early life is associated with reduced longevity (Metcalfe & Monaghan, 2003), which might be due to downstream effects on mitochondrial functionality (Tarry‐Adkins et al., 2016). Here we examined whether blood mitochondrial function (mainly attributed to RBCs; Stier et al., 2013; Dawson & Salmón, 2020): (i) shows age‐related changes within individuals during adult life, (ii) is affected by early‐life growth conditions and (iii) if there is any association between mitochondrial function and survival probability measured into old adulthood.

We experimentally altered the tempo of postnatal growth by inducing catch‐up growth via manipulation of the protein content in the food available during nestling growth using a captive population kept in constant photoperiod and temperature (see Salmón et al 2021; Supporting Information). This experimental approach successfully induce individuals only in the catch‐up growth treatment (birds switched from low‐ to high‐protein diets) to increase their growth rate without generating differences in final body size (Salmón et al., 2021), LH diet group in Figure S1a; post‐independence period: Figure S1b. No growth acceleration was found in the groups maintained on the high‐ or low‐protein diets or in the group that experienced a dietary switch that did not induce catch up growth (birds switched from high‐ to low‐protein diets; Figure S1a).

At two ages during adulthood (36.1 ± 1.6 weeks, hereinafter 36 weeks, and 90.8 ± 4.6 weeks, hereinafter 91 weeks), we measured RBC mitochondrial respiration rates and flux control ratio using the same substrate–uncoupler–inhibitor methodology for intact cells as we described previously (i.e., *ROUTINE*, *LEAK*, *OXPHOS* and *OxCE* see Dawson & Salmón, 2020; Figure 1 and Supporting Information). Mitochondrial respiration rates per unit volume of RBC were normalised for citrate synthase activity (CS), a mitochondrial matrix enzyme often used as a functional marker of mitochondrial content (Larsen et al., 2012). We found that the measured *OXPHOS* coupling efficiency (*OxCE*) decreased with age (Figure 1a; Table S2), suggesting a decline in oxidative phosphorylation, which might lead to a decline in ATP synthesis. Mitochondrial capacity to synthesise ATP has previously been shown to diminish with age in several tissues, including skeletal and cardiac muscle in humans and laboratory rodents (Hebert et al., 2010; Johnson et al., 2013). Our results demonstrate that, in RBCs, a decline in mitochondrial function occurs at the individual level and this is detectable from young to mid‐adulthood. This change in *OxCE* is likely due to an increase in *LEAK* (O_2_ consumption not tied to ATP production) as well as a decrease in *ROUTINE* (mitochondrial respiration of intact RBCs with their endogenous substrates; Figure 1a). Previously, we suggested, that in senescent birds (approx. 6 years old), there might be an increase in mitochondrial RBC volume, inferred by CS‐activity, to compensate for a decline in respiratory capacity (Dawson & Salmón, 2020). Similarly, here, we found an age‐related increase in CS‐activity at middle age (Figure S2d; Table S2); however, the age‐specific mitochondrial respiration rate differences persisted after CS normalisation (Figure 1a). Nonetheless, the decline in *ROUTINE* was only apparent when we normalised the respiration rates to CS activity (Figure S2a). This result suggests that, at the mitochondrial level, there are individual differences in strategy, to sustain the same cellular respiratory capacity, despite the observed overall decline in endogenous respiratory rate (Koch et al., 2021). We also found a significant within individual repeatability (i.e., the proportion of observed variance in a trait attributable to among‐individual differences) in CS‐activity, and, after CS normalisation, in the respiration rates *ROUTINE* and *OXPHOS* (mitochondrial respiration linked to ATP synthesis; Figure 1b). This result suggests that for those mitochondrial traits, individuals maintain their relative rank over successive measurements, demonstrating the consistency of mitochondrial phenotypes over time. There is little information on the consistency of among‐individual differences in mitochondrial function during ageing (Stier et al., 2019), and to our knowledge this is the first time that within individual changes have been evaluated over a considerable time frame, approx. a third of the species typical lifespan: 5–6 years (Rønning et al., 2014).

**FIGURE 1:**
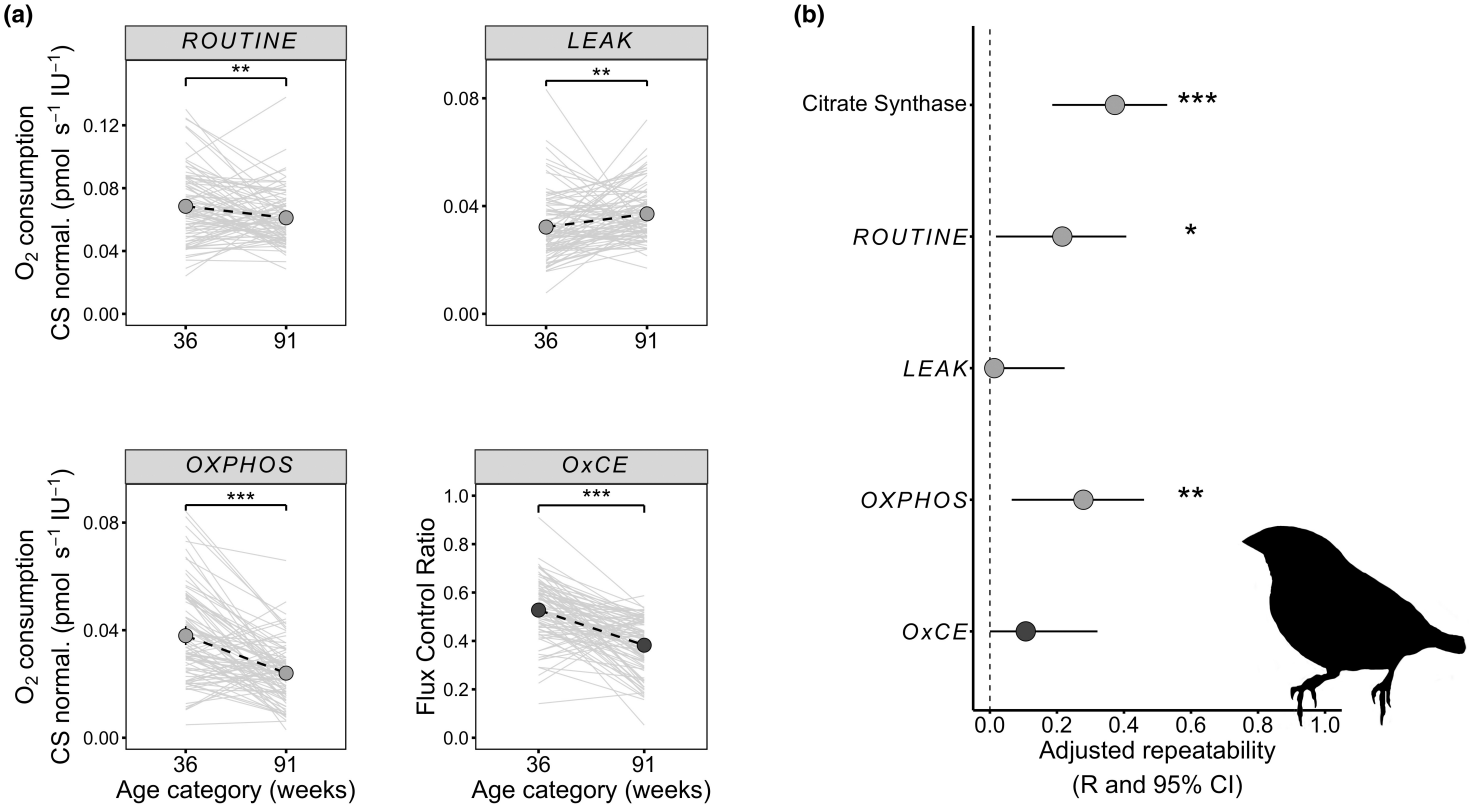
(a) Within individual change with age in mitochondrial respiration rate and flux control ratio in intact red blood cells in adult zebra finches. The panel shows mitochondrial respiration states normalised per unit of citrate synthase activity (CS). No differences were observed in relation to the early life growth manipulation (see text and Supporting Information) and data is pooled by age. (b) Within individual adjusted repeatability of mitochondrial respiration rates CS‐normalised, citrate synthase and *OxCE* between 36 and 91 weeks. *ROUTINE* (*n*_36 /91_ = 91/89 ind.): mitochondrial respiration of intact RBCs with their endogenous substrates; *LEAK* (*n*_36 /91_ = 90/89 ind.): mitochondrial respiration linked to mitochondrial proton leak (obtained after the addition of oligomycin); *OXPHOS* (*n*_36 /91_ = 91/89 ind.): Mitochondrial respiration linked to ATP synthesis (calculated as the difference between *LEAK* and *ROUTINE* respiration); *OxCE* (*n*_36/91_ = 90/89 ind.): apparent OXPHOS coupling efficiency, expressed as a ratio. Data presented as means ± 95%CI. Background grey lines show individuals' trajectory. * *p <* 0.05, **p < 0.01, ***p < 0.001.

Despite the significant age‐related changes in RBC mitochondrial function, we did not find any differences relating to early‐life growth rates following dietary manipulation. This result was consistent for the mitochondrial rates (per unit volume of RBC or normalised by CS: Tables S1 and S2), the *OxCE* and CS‐activity (Table S3). In zebra finches, postnatal growth has been linked to nestling RBCs mitochondrial function (Udino et al., 2021) and growth acceleration is associated with higher whole‐organism metabolic rate in adulthood (Criscuolo et al., 2008). Therefore, we examined whether variation in individual growth rate within the treatment groups was associated with changes in RBC mitochondrial function, but there was no significant pattern observed (all *p* > 0.093; Figure S3). It is plausible that developmental programming of mitochondria, including in RBCs, is only possible during earlier developmental stages (Gyllenhammer et al., 2020; Stier et al., 2022); that the effects do not manifest themselves until the birds reach an older age than those used in our study; or that mitochondrial dysfunction during ageing is likely to be tissue‐specific, as previously reported in flight muscle and liver mitochondria from zebra finches (Salmón, Millet, et al., 2023).

Finally, we evaluated the relationship between RBC mitochondrial function and survival probability (up to 4 years; median: 4, range of monitoring: 3.7–4 years). An age‐related increase in mortality is evident by 3 years of age in zebra finches, and reproductive performance also declines by this age, indicative of an ageing phenotype (Marasco et al., 2018). However, none of the analysed mitochondrial RBC traits correlated with survival probability over this time frame (Figure S4; Table S4). Although the maintenance of mitochondrial function is potentially a key aspect protecting against the ageing process (Brand, 2000), we cannot exclude the possibility that RBC mitochondrial function might not reflect the functionality in other tissues, for example, flight muscle, or that a relationship between mitochondrial function and survival might appear only much later in life (Stier et al., 2021).

In summary, our results demonstrate that within individual age‐related changes in mitochondrial function can be detected using blood cells in birds and these are already evident by mid‐adulthood. This opens new avenues of research in mitochondrial function via the use of tissues that require minimally invasive collection in non‐mammalian vertebrates and reinforces the utility of less conventional animal models in ageing research, in particular birds (Austad, 2011).

## AUTHOR CONTRIBUTIONS

P.S, P.M. and C.S. designed the study; P.S., C.M. and N.D. collected the samples and analysed them; P.S. did the statistical analyses with input from P.M. and C.S.; P.S. wrote the first draft of the manuscript with input from P.M. and C.S.; all authors subsequently contributed to the manuscript.

## CONFLICT OF INTEREST STATEMENT

None declared.

## Supporting information


Data S1:

## Data Availability

Data is available in the Dryad Digital Repository DOI: https://datadryad.org/stash/share/PZPE40yPenGH2WXNsr3Ve9XbSHDC7yTqc732PCVXbZM (Salmón, Dawson et al., 2023).
