# Supplementary material for: Mitochondrial function declines with age within individuals but is not linked to the pattern of growth or mortality risk in zebra finch
Source: Aging Cell. 2023 Mar 20;22(6):e13822. doi: 10.1111/acel.13822 (PMC10265141; doi:10.1111/acel.13822)
Supplement: Supplementary file 1 — Data S1: [file ACEL-22-e13822-s001.docx]

**Supporting Information:**

Mitochondrial function declines with age within individuals but is not linked to the pattern of growth or mortality risk in zebra finch

**Authors:** Pablo Salmón^1,2^, Neal J. Dawson^1^, Caroline Millet^1^, Colin Selman^1^ and Pat Monaghan^1^

^1^School of Biodiversity, One Health and Veterinary Medicine, University of Glasgow, Glasgow, UK

^2^Institute of Avian Research “Vogelwarte Helgoland”, Wilhelmshaven, Germany

**Correspondence:** Pablo Salmón, Institute of Avian Research “Vogelwarte Helgoland”, An der Vogelwarte 21, Wilhelmshaven 26386, Germany. Email: [pablo.salmon@ifv-vogelwarte.de](mailto:pablo.salmon@ifv-vogelwarte.de)

**Experimental Procedures**

***Experimental design and blood sampling***

We sampled adult zebra finches at two time points, 36 weeks (mean: 36.1, median: 36, range: 32-39) and 91 weeks (mean: 90.8, median: 90, range: 72-100). The early life conditions of these birds (hatching to 15 days) were manipulated to induce growth acceleration during the period of life when most body growth is happening (see Salmón et al. 2021 for details). Briefly, we fed diets high (H) and low (L) in protein content to induce differences in growth rates. The H diet consisted of 5 g of protein conditioning supplement (J.E. Haith, Cleethorpes, UK, 13.6% protein), a daily addition of 5 g of homogenized hard-boiled hens’ egg, with mixed seeds and water *ad libitum*. The L diet comprised only of mixed seeds and water *ad libitum* (Birkhead et al. 1999; Blount et al. 2003; Criscuolo et al. 2008). Weekly fresh spinach, Calvicet calcium and water-soluble vitamin supplement was provided in both diets. Birds were maintained throughout the study under a photoperiod of 14h:10h (light: dark cycle) and constant ambient temperature (20-24 °C). Overall, the main difference between the two experimental diets was their final protein content, which comprised approximately 40% in the H diet and 12% in the L diet (Criscuolo et al. 2008; Salmón et al. 2021)

The manipulation was done at the family level since the parents carry food to their young. However, by reducing the quality of the food, we made it harder for parents to compensate by simply increasing the volume of food provided since this was provided *ad libitum*. There were 2 phases to the manipulation. The 1^st^ phase lasted for the first 7 days from hatching of the first chick, and nests were assigned to either the H or L diet throughout this phase. When at least half of the nestlings within a brood reached 7 days of age (median: 7 days, range: 5-9 days), the whole brood was again randomly allocated to their 2^nd^ phase diet, either H or L-quality until 15 days of age (median: 15 days, range: 13-18 days), thereby creating four different experimental groups i.e. HH (High-High), HL (High-Low), LL (Low-Low) and LH (Low-High) according to the diet they were exposed to during the 1^st^ and 2^nd^ phase of the experimental manipulation. These four groups were designed to manipulate the individuals’ growth trajectory and to separate the effects of the two diets *per se* from the diet switching; HH and LL individuals had high and low protein diets throughout, while HL and LH both experienced a switch in diet. Growth acceleration, relative to the other groups, during the main growth phase was expected only in the LH group, following the switch from a low to a high protein diet at the start of this period (at 7 days). The LL group was predicted to grow slower than the other groups during the dietary manipulation period since they always had a low protein diet.

From 15 days onwards, the same diet was provided to all families consisting of *ad libitum* mixed seed- common millet, yellow millet and canary seed in a ratio 3:1:1 (Johnson and Jeff, Gilberdyke, UK)- and cuttlefish (this was termed pre-hatching parental diet), with *ad libitum* access to water. Nestlings were separated from their parents at 30 days of age (median: 30 days, range: 28-33 days) and transferred into mixed sex “fledgling flocks” composed of individuals from multiple families (typically 6-8 individuals per flock). At 50 days of age (median: 50 days, range: 47-55), sex was determined by plumage characteristics, enabling birds to be subsequently transferred into unisex aviaries (60 individuals per aviary). All housing conditions and experimental protocols were approved by and carried out under Home Office Project Licence (70/8335), following local ethical review.

At hatching, nestlings were given identifying ink marks on their toenails, and their body mass (± 0.01 g) and tarsus length were recorded. Body mass and tarsus length were again recorded at 7 days of age (switch of diet), 15 days (end of manipulation), 30 days (parental separation), 50 days, 120 days (median: 121 days, range: 117-124), 250 days (ca. 36 weeks), 365 days (median: 381 days, range: 351-394) and 635 days (ca. 91 weeks). For this study a blood sample (~ 120-140 µl) was collected at 36 and 91 weeks from the brachial vein using a heparinised capillary tube within 5 min after capture and stored on ice until further processing. Samples were then centrifuged at 3,000 g for 10 min at 4°C to separate plasma from cell pellet. The plasma was removed, and the pellet was gently resuspended in 1 ml μl of ice-cold respiration medium (MiR05, Gnaiger et al 2000 [0.5 mM EGTA, 3 mM MgCl_2_, 60 mM K-lactobionate, 20 mM taurine, 10 mM KH_2_PO_4_, 20 mM Hepes, 110 mM sucrose, free fatty acid bovine serum albumin (1 g l^-1^), pH 7.1]). The resuspended samples were centrifuged again at 3,000 g for 5 min and the supernatant discarded.

A total of 92 individuals from 40 families (corresponding to the first batch in Salmón et al 2021) and that survived until the 36 weeks of age were used in this study (individuals/family per final experimental group: 23/10 HH; 21/10 HL; 26/10 LL and 23/10 LH). The observed mortality rate during the early life experimental manipulation was approximately 6.5%, and comparable to previous studies using wild and domestic zebra finch populations (Tschirren et al. 2009) and no differences were observed between treatments (Salmón et al. 2021).

***Mitochondrial respiration in intact blood cells***

Mitochondrial respiration was performed using previously described methods for intact avian blood cells (Dawson & Salmón 2020; Nord et al., 2021; Stier et al. 2017). After the above-described centrifugation steps and within 30 min since obtaining the blood sample, 40 μl were pippeted from the bottom of the cell pellet, mainly composed by red blood cells- hereinafter RBCs- (Samour 2006), and resuspended in 1 ml of MiR05 respiration medium incubated at 41°C. The RBCs resuspension was added to an additional 1 ml of respiration medium in the respirometry chamber of an Oxygraph-2K high resolution respirometer (Oroboros Instruments, Innsbruck, Austria). After addition of the RBCs, the chamber was kept open for a minute to allow for oxygen re-equilibration. Respiration rate was measured as the rate of decline in O_2_ concentration in the chamber. The chamber was then closed and the O_2_ consumption signal was allowed to stabilize (*ROUTINE*; mitochondrial respiration of intact RBCs with their endogenous substrates). We then added 2.5 μM of oligomycin for 2-3 minutes in order to inhibit mitochondrial ATP synthesis *(LEAK*; mitochondrial respiration linked to mitochondrial proton leak), followed by 5 μM of antimycin A to account for residual or non-mitochondrial oxygen consumption. Residual oxygen consumption was subtracted from all respiration states. Mitochondrial respiration linked to oxidative phosphorylation (*OXPHOS*) was calculated by subtracting *LEAK* from *ROUTINE*. In addition, we calculated an apparent index, as transmembrane potential could vary across respiration states and hence *LEAK* respiration, of OXPHOS coupling efficiency (*OxCE*), which is a flux control ratio (1 - *LEAK/ROUTINE*; see Ton et al 2021). Due to the limited volume of RBCs, samples were run in singlet, as this protocol has been shown to be technically highly repeatable (Stier et al., 2017), and the intra-class coefficient of correlation (ICC) based on duplicates in a subset of samples (n=17) corroborated this for our study *ROUTINE* (ICC = 0.89, 95% CI [0.72-0.96], *p<0.001*), *LEAK* (ICC= 0.92, 95% CI [0.80-0.97], p<0.001), *OXPHOS* (ICC = 0.84, 95% CI [0.60-0.94], *p<0.001*) and *OxCE* (ICC = 0.89, 95% CI [0.72-0.96], *p<0.001*). All chemicals were purchased from Sigma-Aldrich (UK).

***Citrate synthase content measurement***

The maximal activity of citrate synthase (CS), a commonly used marker for normalising mitochondrial respiration rates (e.g., Larsen et al. 2012), was assayed in RBCs at avian body temperature (41°C) as described in (Dawson & Salmón 2020). Samples were kept on ice and homogenized in 2.5 volumes of homogenizing buffer [100 mmol l^−1^ KH_2_PO_4_ buffer, pH 7.2, containing 1 mmol l^−1^ EGTA, 1 mmol l^−1^ EDTA and 1 mmol l^−1^ phenylmethylsulfonyl fluoride (PMSF)]. Homogenates were then freeze at -80°C for 20 min, and thawed, before individually mixed using a Dounce homogeniser at 100 rpm (Cole-Parmer PTFE Tissue Grinder, Cambridshire, UK) for 3 up-down cycles (staying for 30s on ice in between) and finally centrifuged at 2,000 g at 4°C for 5 min. The pellet was discarded, and the collected supernatant was stored at -80°C until used in the assay. Enzyme activity was measured in triplicates and the background activity was subtracted from the final value. Measurements were carried out at 412 nm [ε=14.15 (mmol l^−1^)^−1^] in 100  mmol l^−1^ KH_2_PO_4_ (pH 7.2), 0.15 mmol l^−1^ acetyl-coA, 0.15 mmol l^−1^ 5,5′-dithiobis-2-nitrobenzoic acid, and 0.5 mmol l−1 oxoaloacetate (omitted in blank). The assay was run using a SpectraMaxPlus 384 spectrophotometer (Molecular Devices), and data were analysed using the accompanying SoftMax Pro 6.3 program. The intra-assay technical repeatability of the triplicates was high (ICC = 0.92, 95% CI [0.91-0.94], *p<0.001*) and inter-assay coefficient of variation was low (CV: 4.4% based on a pooled sample across assays, n=8). Mean values were used in all analyses. Protein concentrations were determined using the Bradford method (Bio-Rad, UK) and assayed in triplicate (ICC = 0.93, 95% CI [0.74-0.97], *p<0.001*). There were no differences in RBCs protein levels per μl between 36 and 91 weeks (F_1,88.10_ = 2.49, p=0.118).

***Statistical analyses***

We first examined the effect of the experimental manipulation on the body mass trajectory in two independent models, one from hatching to 30 days (prior to independence) and another from 50 to 91 weeks (after independence). In both cases we used linear mixed-effects models with Gaussian error distribution with the 1^st^ phase (two level factor: High -H - or Low -L -), the 2^nd^ phase (7-15 days two level factor: H or L), age category and their 3-way interaction. The models also included individuals’ sex as fixed effect and tarsus length as a covariate. In order to account for the variation in actual age at which individuals were measured at each sampling point hatching rank (two level factor: 1^st^ or 2^nd^) was included as a factor in the model prior-to-independence and individuals’ exact age in the after-independence model. The nest ID (family) and individuals’ ID as random effects. Body mass, tarsus length and individuals’ exact age were Z-transformed to have mean = 0 and SD = 1.

We then analysed the effects of age on mitochondrial respiration metrics normalised to CS activity, i.e., *ROUTINE*, *LEAK*, *OXPHOS*; CS activity; and the flux control ratio (*OxCE*). Each model included 1st phase diet, 2nd phase diet, age category (two level factor: 36 weeks and 91 weeks), sex and hatching rank as fixed effects and the nest ID and individuals’ ID as random effects. The triple interaction was also initially included in all models “*1^st^ phase x 2^nd^ phase x age category*” to explore if the early life manipulation influenced the mitochondrial respiration change. Previous work suggest potential circadian variations in mitochondrial function (Stier et al. 2022; van Moorsel et al. 2016), however, an initial inspection on that respect in our data indicates no overall effect of the time of the day (all p>0.05); thus, to avoid overparameterization in our models we did not include time as a covariate. In addition, we explored the stage-dependent relationship between the rate of growth (measured as g d^-1^) during the main postnatal growth phase, i.e., 7 to 15 days (see Salmón et al. 2021), and the adulthood mitochondrial respiration metrics and *OxCE* at 36- and 91-weeks using Pearson’s correlations. The sample size for these analyses consisted in 180 samples from 92 individuals (36_weeks_= 91, 91_weeks_= 89; 3 individuals died between sampling periods and in one individual there was not enough RBCs’ volume).

We calculate the intra-individual adjusted repeatability of mitochondrial respiration rates (per unit volume of RBC and CS-normalised), CS activity and *OxCE* using the R package *rptr* (Stoffel, Nakagawa, & Schielzeth, [2017](https://onlinelibrary.wiley.com/doi/full/10.1111/mec.15331#mec15331-bib-0060)) with the 95% confidence interval (95% CI) being obtained from 1,000 bootstrap iterations. Each model was fitted with a normal error distribution and included the fixed effect of age category and random effects of individuals’ and family IDs. Finally, mortality (26 cases) was recorded daily and was analysed using Cox proportional hazards models using the R package *coxme* (Therneau & Therneau 2015), with numbers of days from sampling till death as the dependent variable. Birds still alive on 7^th^ April 2022 were censored (1,4621.14 ± 17.73 days old; median: 1,466; range: 1,360-1,482). We fit independent models with each of the CS- normalised mitochondrial respiration rates, CS-activity or *OxCE* as covariate together with sex, as sex-specific mortality has previously been observed during the studied ages in our populations (Monaghan et al. 2012)*.* In addition, we include family ID and bird ID as random factors. No violation of the proportionally assumption was observed in any of the models (tested using Schoenfeld residuals).

All statistical analyses were performed in R 3.5.2 (R Core Team 2018) and the significance of parameter estimates was estimated using F-tests based on Satterthwaite approximation for the denominator degrees of freedom. In all cases, the distribution of residuals was inspected visually and did not show marked deviations from normality. Finals models were achieved by omitting non-significant interaction terms only (p > 0.05 when comparing a reduced model to the original model in likelihood ratio tests), retaining all main effects even if not significant. Significant interactions were further explored using pairwise planned comparisons adjusted by Tukey HSD in *emmeans* (Lenth et al. 2018). The explanatory power of the final models was calculated as marginal (R^2^_m_) and conditional (R^2^_c_) values following (Nakagawa & Schielzeth 2013) and implemented in *r2glmm* (Jaeger et al. 2017).

***References***

Birkhead TR, Fletcher F & Pellatt EJ (1999) Nestling diet, secondary sexual traits and fitness in the zebra finch. Proceedings of the Royal Society of London. Series B: Biological Sciences 266, 385–390.

Blount JD, Metcalfe NB, Arnold KE, Surai PF, Devevey GL & Monaghan P (2003) Neonatal nutrition, adult antioxidant defences and sexual attractiveness in the zebra finch. Proc Biol Sci 270, 1691–1696.

Criscuolo F, Monaghan P, Nasir L & Metcalfe NB (2008) Early nutrition and phenotypic development: ‘catch-up’ growth leads to elevated metabolic rate in adulthood. Proceedings of the Royal Society B: Biological Sciences 275, 1565–1570.

Dawson NJ & Salmón P (2020) Age-related increase in mitochondrial quantity may mitigate a decline in mitochondrial quality in red blood cells from zebra finches (Taeniopygia guttata). Exp. Gerontol. 133, 110883.

Gnaiger E, Kuznetsov AV, Schneeberger S, Seiler R, Brandacher G, Steurer W, Margreiter R (2000) Mitochondria in the cold. In: Heldmaier G, Klingenspor M (eds) Life in the cold. Springer, Heiderlberg, Berlin, New York, pp 431–442

Jaeger BC, Edwards LJ, Das K & Sen PK (2017) An R2 statistic for fixed effects in the generalized linear mixed model. Journal of Applied Statistics 44, 1086–1105.

Larsen S, Nielsen J, Hansen CN, Nielsen LB, Wibrand F, Stride N, Schroder HD, Boushel R, Helge JW, Dela F & Hey-Mogensen M (2012) Biomarkers of mitochondrial content in skeletal muscle of healthy young human subjects. J. Physiol. (Lond.) 590, 3349–3360.

Lenth R, Singmann H, Love J, Buerkner P & Herve M (2018) Emmeans: Estimated marginal means, aka least-squares means. R package version 1.4.7 1, 3.

Monaghan P, Heidinger BJ, D’Alba L, Evans NP & Spencer KA (2012) For better or worse: reduced adult lifespan following early-life stress is transmitted to breeding partners. Proceedings of the Royal Society B: Biological Sciences 279, 709–714.

van Moorsel D, Hansen J, Havekes B, Scheer FAJL, Jörgensen JA, Hoeks J, Schrauwen-Hinderling VB, Duez H, Lefebvre P, Schaper NC, Hesselink MKC, Staels B & Schrauwen P (2016) Demonstration of a day-night rhythm in human skeletal muscle oxidative capacity. Mol Metab 5, 635–645.

Nakagawa S & Schielzeth H (2013) A general and simple method for obtaining R2 from generalized linear mixed-effects models. Methods in Ecology and Evolution 4, 133–142.

Nord, A., Metcalfe, N. B., Page, J. L., Huxtable, A., McCafferty, D. J., & Dawson, N. J. (2021). Avian red blood cell mitochondria produce more heat in winter than in autumn. The FASEB Journal, 35(5), e21490.

R Core Team (2018) R: A language and environment for statistical computing. R Foundation for Statistical Computing, Vienna, Austria.

Salmón P, Millet C, Selman C & Monaghan P (2021) Growth acceleration results in faster telomere shortening later in life. Proceedings of the Royal Society B: Biological Sciences 288, 20211118.

Samour, J. (2006). Diagnostic value of hematology. Clinical avian medicine, 2, 587-609.

Stier, A., Monaghan, P., & Metcalfe, N. B. (2022). Experimental demonstration of prenatal programming of mitochondrial aerobic metabolism lasting until adulthood. Proceedings of the Royal Society B, 289(1970), 20212679.

Stier A, Romestaing C, Schull Q, Lefol E, Robin J-P, Roussel D & Bize P (2017) How to measure mitochondrial function in birds using red blood cells: a case study in the king penguin and perspectives in ecology and evolution. Methods in Ecology and Evolution 8, 1172–1182.

Ton, R., Stier, A., Cooper, C. E., & Griffith, S. C. (2021). Effects of heat waves during post-natal development on mitochondrial and whole body physiology: an experimental study in zebra finches. Frontiers in Physiology, 554.

Therneau TM & Therneau MTM (2015) Package ‘coxme.’ R package version 2.

Tschirren B, Rutstein AN, Postma E, Mariette M & Griffith SC (2009) Short- and long-term consequences of early developmental conditions: a case study on wild and domesticated zebra finches. J. Evol. Biol. 22, 387–395.

**Figure S1.** Postnatal body mass trajectory from hatching to 91 weeks (630 days) of zebra finch undergoing an experimental manipulation of their early life growth trajectory (see methods for details). *(a)* prior independence; *(b)* post-independence. Experimental groups are coded as 1^st^ phase group - 2^nd^ phase group, where H= High, L= Low. H: grey; L: white; HH: red; HL: blue; LL: yellow and LH: grey. The horizontal bar in “*a*” indicates the experimental period, the group induced to accelerate growth is LH and is highlighted with a dotted line. In “*a*” n.s.: p>0.05 and ***: *p<0.001* indicate Tukey HSD post-hoc comparisons between experimental groups as the 3-way interaction: ”*1^st^ phase group x 2^nd^ phase group x age category*” was significant (F_3, 263.89_= 9.26, *p<0.001*). In “*b*” the interaction was not significant (F_4, 343.47_= 0.62, p=0.650) nor the 2-way interactions: “*1^st^ phase group x age category”* or *“2^nd^ phase group x age category”* (all p>0.077). Data presented as means ± sem, points are jittered for visual purposes and background grey plotting show raw data.

**Figure S2.** Age variation in mitochondrial respiration in intact red blood cells (mitochondrial rates per unit volume of RBC) and citrate synthase activity in adult zebra finches exposed to an early life growth manipulation. The panel shows *(a)* baseline (*ROUTINE)*, mitochondrial respiration of intact RBCs with their endogenous substrates; *(b)* *LEAK*, mitochondrial respiration linked to mitochondrial proton leak; and *(c)* Mitochondrial respiration linked to ATP synthesis (*OXPHOS*); and *(d)* Citrate synthase activity, a marker of mitochondrial volume per unit of volume of RBC. Note there were no significant differences between early life experimental groups and the data is pooled. Data presented as means ± 95%CI. Background grey lines show individuals’ trajectory. n.s. p>0.05, *** *p<0.001*

**Figure S3.** Age-dependent Pearson correlation coefficients between red blood cells mitochondrial respiration rates (*ROUTINE*, *LEAK* and *OXPHOS*) normalised by citrate synthase, Flux Control Ratio (*OxCE*) and citrate synthase activity and mass growth rate (g d^-1^) between 7- and 15-days post hatching (period when growth manipulation was performed; in LH group showed growth acceleration, see Figure S2). The data is grouped by experimental groups for visualisation purposes. No significant differences were observed among them or when all groups were pooled – “*All”*- (r= -0.34 to 0.25, 95% [-0.65 to -0.11, 0.06 to 0.78]). Experimental groups coded as 1^st^ phase group - 2^nd^ phase group, where H= High, L= Low. HH: red; HL: blue; LL: yellow and LH: grey; All: white, represents the value for all the groups together.

**Figure S4.** None of the mitochondrial respiration rates (normalised by citrate synthase) or the Flux Control Ratio (*OxCE*) were associated with survival probability up to April 2022 (i.e., approx. 4 years of age). For illustrative purposes, the mitochondrial rates and *OxCE* are grouped by three quartiles (lowest: <25%, dashed; the two middles: 25%-75%, dotted; and highest: >75%, solid). Note however that we performed the analyses with them as continuous variables.

**Table S1.** Summary of models testing the influence of age and early life treatment in mitochondrial respiration in intact red blood cells (per unit volume of red blood cell) in zebra finches: (a) baseline (*ROUTINE*), mitochondrial respiration of intact RBCs with their endogenous substrates; (b) *LEAK*, mitochondrial respiration linked to mitochondrial proton leak; and (c) Mitochondrial respiration linked to ATP synthesis (*OXPHOS*). Early life experimental manipulation: 1^st^ phase (0-7 days of age), 2^nd^ phase (7-15 days of age), L= Low quality diet, H= High quality diet. All models include nest ID (family) and gel ID as random effects. Rejected terms are in italics and final models’ explanatory power is represented as marginal (R^2^_m_) and conditional (R^2^_c_) values. In all models, bird ID and family ID are included as random effects. Italics font indicate terms removed from the final model.

|  | Estimate | SE | df | F | p-value |
| --- | --- | --- | --- | --- | --- |
| (a) Dependent variable: *ROUTINE* resp. |  |  |  |  |  |
| (Intercept) | 3.44 x10^-01^ | 1.25 x10^-02^ |  |  |  |
| Age category (91 weeks) | 1.61 x10^-02^ | 9.66 x10^-03^ | 1, 90.26 | 2.77 | 0.100 |
| 1^st^ phase (L) | 1.45 x10^-02^ | 1.05 x10^-02^ | 1, 86.33 | 1.90 | 0.172 |
| 2^nd^ phase (L) | 1.44 x10^-02^ | 1.03 x10^-02^ | 1, 86.53 | 0.02 | 0.890 |
| Sex (M) | -2.22 x10^-02^ | 1.04 x10^-02^ | 1, 86.47 | 4.55 | **0.036** |
| Hatching rank (2^nd^) | 1.17 x10^-02^ | 1.09 x10^-02^ | 1, 87.03 | 1.16 | 0.284 |
| *Age category (91 weeks) x 1^st^ phase (L)* | *-1.79 x10^-02^* | *2.73 x10^-02^* | *1, 87.40* | *0.03* | *0.865* |
| *Age category (91 weeks) x 2^nd^ phase (L)* | *-4.02 x10^-02^* | *2.85 x10^-02^* | *1, 87.40* | *0.94* | *0.334* |
| *1^st^ phase (L) x 2^nd^ phase (L)* | *-3.18 x10^-02^* | *2.86 x10^-02^* | *1, 86.24* | *0.25* | *0.616* |
| *Age category (91 weeks) x 1^st^ phase (L) x 2^nd^ phase (L)* | *4.24 x10^-02^* | *3.90 x10^-02^* | *1, 87.40* | *1.18* | *0.280* |
| R^2^_m_/ R^2^_c_= 0.06/ 0.12 |  |  |  |  |  |
|  |  |  |  |  |  |
| (b) Dependent variable: *LEAK* resp. |  |  |  |  |  |
| (Intercept) | 1.68 x10^-01^ | 8.40 x10^-03^ |  |  |  |
| Age category (91 weeks) | 6.11 x10^-02^ | 6.39 x10^-03^ | 1, 90.81 | 91.45 | **<0.001** |
| 1^st^ phase (L) | -3.94 x10^-03^ | 6.98 x10^-03^ | 1, 87.25 | 0.32 | 0.574 |
| 2^nd^ phase (L) | 3.00 x10^-04^ | 6.85 x10^-03^ | 1, 87.03 | 0.00 | 0.965 |
| Sex (M) | -1.29 x10^-02^ | 6.90 x10^-03^ | 1, 87.26 | 3.48 | 0.065 |
| Hatching rank (2^nd^) | 2.24 x10^-03^ | 7.19 x10^-03^ | 1, 87.49 | 0.10 | 0.756 |
| *Age category (91 weeks) x 1^st^ phase (L)* | *1.30 x10^-02^* | *1.78 x10^-02^* | *1, 91.78* | *0.04* | *0.848* |
| *Age category (91 weeks) x 2^nd^ phase (L)* | *9.17 x10^-03^* | *1.86 x10^-02^* | *1, 91.78* | *0.01* | *0.914* |
| *1^st^ phase (L) x 2^nd^ phase (L)* | *-1.17 x10^-03^* | *1.85 x10^-02^* | *1, 92.55* | *0.76* | *0.385* |
| *Age category (91 weeks) x 1^st^ phase (L) x 2^nd^ phase (L)* | *-2.11E x10^-02^* | *2.54 x10^-02^* | *1, 91.78* | *0.69* | *0.409* |
| R^2^_m_/ R^2^_c_= 0.33/ 0.38 |  |  |  |  |  |
|  |  |  |  |  |  |
| (c) Dependent variable: *OXPHOS* resp. |  |  |  |  |  |
| (Intercept) | -4.83 x10^-02^ | 8.61 x10^-03^ |  |  |  |
| Age category (91 weeks) | 1.44 x10^-02^ | 9.73 x10^-03^ | 89.02 | 31.39 | **<0.001** |
| 1^st^ phase (L) | -1.55 x10^-03^ | 9.57 x10^-03^ | 85.24 | 2.20 | 0.142 |
| 2^nd^ phase (L) | -1.28 x10^-02^ | 9.63 x10^-03^ | 85.44 | 0.03 | 0.872 |
| Sex (M) | 6.61 x10^-03^ | 1.00 x10^-02^ | 85.39 | 1.78 | 0.186 |
| Hatching rank (2^nd^) | -4.83 x10^-02^ | 8.61 x10^-03^ | 85.94 | 0.43 | 0.512 |
| *Age category (91 weeks) x 1^st^ phase (L)* | *-1.82 x10^-02^* | *2.37 x10^-02^* | *1, 89.96* | *0.19* | *0.667* |
| *Age category (91 weeks) x 2^nd^ phase (L)* | *-3.68 x10^-02^* | *2.48 x10^-02^* | *1, 89.96* | *0.44* | *0.509* |
| *1^st^ phase (L) x 2^nd^ phase (L)* | *-1.89 x10^-02^* | *2.52 x10^-02^* | *1, 90.95* | *0.13* | *0.723* |
| *Age category (91 weeks) x 1^st^ phase (L) x 2^nd^ phase (L)* | *5.11 x10^-02^* | *3.39 x10^-02^* | *1, 89.96* | *2.27* | *0.136* |
| R^2^_m_/ R^2^_c_= 0.16/ 0.25 |  |  |  |  |  |

**Table S2.** Summary of models testing the influence of age and early life treatment in zebra finch red blood cells mitochondrial: (a) the flux control ratio*, OxCE*, an apparent index of OXPHOS coupling efficiency; (b) Citrate synthase activity per μl of RBC. Early life experimental manipulation: 1^st^ phase (0-7 days of age), 2^nd^ phase (7-15 days of age), L= Low quality diet, H= High quality diet. All models include nest ID (family) and gel ID as random effects. Rejected terms are in italics and final models’ explanatory power is represented as marginal (R^2^_m_) and conditional (R^2^_c_) values. In all models, bird ID and family ID are included as random effects. Italics font indicate terms removed from the final model.

|  | Estimate | SE | df | F | p-value |
| --- | --- | --- | --- | --- | --- |
| (a) Dependent variable: *OxCE* |  |  |  |  |  |
| (Intercept) | 5.14 x10^-01^ | 2.38 x10^-02^ |  |  |  |
| Age category (91 weeks) | -1.45 x10^-01^ | 1.74 x10^-02^ | 1, 89.15 | 69.17 | **<0.001** |
| 1^st^ phase (L) | 2.95 x10^-02^ | 1.99 x10^-02^ | 1, 85.83 | 2.20 | 0.142 |
| 2^nd^ phase (L) | -6.52 x10^-03^ | 1.95 x10^-02^ | 1, 85.62 | 0.11 | 0.739 |
| Sex (M) | -1.94 x10^-03^ | 1.97 x10^-02^ | 1, 85.84 | 0.01 | 0.922 |
| Hatching rank (2^nd^) | 6.08 x10^-03^ | 2.05 x10^-02^ | 1, 86.08 | 0.09 | 0.768 |
| *Age category (91 weeks) x 1^st^ phase (L)* | *-4.24 x10^-02^* | *4.91 x10^-02^* | *1, 85.97* | *0.24* | *0.626* |
| *Age category (91 weeks) x 2^nd^ phase (L)* | *-7.83 x10^-02^* | *5.12 x10^-02^* | *1, 85.97* | *0.29* | *0.594* |
| *1^st^ phase (L) x 2^nd^ phase (L)* | *-3.16 x10^-02^* | *5.27 x10^-02^* | *1, 85.06* | *0.50* | *0.482* |
| *Age category (91 weeks) x 1^st^ phase (L) x 2^nd^ phase (L)* | *1.19 x10^-01^* | *6.99 x10^-02^* | *1, 85.97* | *2.91* | *0.092* |
| R2_m_/ R2_c_= 0.27/ 0.35 |  |  |  |  |  |
|  |  |  |  |  |  |
| (b) Dependent variable: Citrate synthase activity |  |  |  |  |  |
| (Intercept) | 5.48 | 2.81 x10^-01^ |  |  |  |
| Age category (91 weeks) | 8.64 x10^-01^ | 1.67 x10^-01^ | 1, 89.77 | 26.90 | **<0.001** |
| 1^st^ phase (L) | 2.09 x10^-01^ | 2.44 x10^-01^ | 1, 33.67 | 0.74 | 0.397 |
| 2^nd^ phase (L) | 3.00 x10^-01^ | 2.39 x10^-01^ | 1, 34.58 | 1.57 | 0.219 |
| Sex (M) | -7.07 x10^-01^ | 2.33 x10^-01^ | 1, 80.62 | 9.19 | **0.003** |
| Hatching rank (2^nd^) | -1.45 x10^-01^ | 2.43 x10^-01^ | 1, 74.41 | 0.35 | 0.553 |
| *Age category (91 weeks) x 1^st^ phase (L)* | *-1.18 x10^-01^* | *4.73 x10^-01^* | *1, 86.49* | *0.67* | *0.417* |
| *Age category (91 weeks) x 2^nd^ phase (L)* | *7.65 x10^-02^* | *4.94 x10^-01^* | *1, 86.49* | *0.06* | *0.812* |
| *1^st^ phase (L) x 2^nd^ phase (L)* | *-6.20 x10^-01^* | *5.82 x10^-01^* | *1, 36.25* | *2.67* | *0.111* |
| *Age category (91 weeks) x 1^st^ phase (L) x 2^nd^ phase (L)* | *-3.14 x10^-01^* | *6.76 x10^-01^* | *1, 86.48* | *0.22* | *0.643* |
| R^2^_m_/ R^2^_c_= 0.16/ 0.44 |  |  |  |  |  |

**Table S3.** Summary of models testing the influence of age and early life treatment in mitochondrial respiration in intact red blood cells (normalised per citrate synthase activity) in zebra finches: (a) baseline (*ROUTINE*), mitochondrial respiration of intact RBCs with their endogenous substrates; (b) *LEAK*, mitochondrial respiration linked to mitochondrial proton leak; and (c) Mitochondrial respiration linked to ATP synthesis (*OXPHOS*). Early life experimental manipulation: 1^st^ phase (0-7 days of age), 2^nd^ phase (7-15 days of age), L= Low quality diet, H= High quality diet. All models include nest ID (family) and gel ID as random effects. Rejected terms are in italics and final models’ explanatory power is represented as marginal (R^2^_m_) and conditional (R^2^_c_) values. In all models, bird ID and family ID are included as random effects. Italics font indicate terms removed from the final model.

|  | Estimate | SE | df | F | p-value |
| --- | --- | --- | --- | --- | --- |
| (a) Dependent variable: *ROUTINE* (Citrate synthase normalised) |  |  |  |  |  |
| (Intercept) | 0.07 | 4.13 x10^-03^ |  |  |  |
| Age category (91 weeks) | -8.49 x10^-03^ | 2.81 x10^-03^ | 1, 90.63 | 9.15 | **0.003** |
| 1^st^ phase (L) | 1.18 x10^-03^ | 3.54 x10^-03^ | 1, 87.31 | 0.11 | 0.739 |
| 2^nd^ phase (L) | -2.73 x10^-03^ | 3.48 x10^-03^ | 1, 87.50 | 0.61 | 0.436 |
| Sex (M) | 6.08 x10^-03^ | 3.51 x10^-03^ | 1, 87.45 | 3.01 | 0.086 |
| Hatching rank (2^nd^) | 3.12 x10^-03^ | 3.66 x10^-03^ | 1, 87.98 | 0.73 | 0.396 |
| *Age category (91 weeks) x 1^st^ phase (L)* | *-4.08 x10^-04^* | *7.98 x10^-03^* | *1, 87.42* | *0.32* | *0.574* |
| *Age category (91 weeks) x 2^nd^ phase (L)* | *-4.50 x10^-03^* | *8.34 x10^-03^* | *1, 87.42* | *0.02* | *0.878* |
| *1^st^ phase (L) x 2^nd^ phase (L)* | *5.88 x10^-03^* | *9.01 x10^-03^* | *1, 86.79* | *1.84* | *0.179* |
| *Age category (91 weeks) x 1^st^ phase (L) x 2^nd^ phase (L)* | *7.25 x10^-03^* | *1.14 x10^-02^* | *1, 87.42* | *0.40* | *0.527* |
| R^2^_m_/ R^2^_c_= 0.06/ 0.26 |  |  |  |  |  |
|  |  |  |  |  |  |
| (b) Dependent variable: *LEAK* (Citrate synthase normalised) |  |  |  |  |  |
| (Intercept) | 3.20 x10^-02^ | 2.12 x10^-03^ |  |  |  |
| Age category (91 weeks) | 4.85 x10^-03^ | 1.69 x10^-03^ | 1, 91.30 | 8.26 | **0.005** |
| 1^st^ phase (L) | -1.26 x10^-03^ | 1.75 x10^-03^ | 1, 87.47 | 0.52 | 0.473 |
| 2^nd^ phase (L) | -1.35 x10^-03^ | 1.72 x10^-03^ | 1, 87.26 | 0.62 | 0.432 |
| Sex (M) | 2.52 x10^-03^ | 1.73 x10^-03^ | 1, 87.48 | 2.12 | 0.149 |
| Hatching rank (2^nd^) | 6.80 x10^-04^ | 1.80 x10^-03^ | 1, 87.71 | 0.14 | 0.707 |
| *Age category (91 weeks) x 1^st^ phase (L)* | *2.17 x10^-03^* | *4.82 x10^-03^* | *1, 169.00* | *0.08* | *0.773* |
| *Age category (91 weeks) x 2^nd^ phase (L)* | *1.89 x10^-03^* | *5.03 x10^-03^* | *1, 169.00* | *0.04* | *0.836* |
| *1^st^ phase (L) x 2^nd^ phase (L)* | *5.50 x10^-03^* | *4.87 x10^-03^* | *1, 169.00* | *1.56* | *0.214* |
| *Age category (91 weeks) x 1^st^ phase (L) x 2^nd^ phase (L)* | *-2.36 x10^-03^* | *6.87 x10^-03^* | *1, 169.00* | *0.12* | *0.732* |
| R^2^_m_/ R^2^_c_= 0.06/ 0.08 |  |  |  |  |  |
|  |  |  |  |  |  |
| (c) Dependent variable: *OXPHOS* (Citrate synthase normalised) |  |  |  |  |  |
| (Intercept) | 3.59 x10^-02^ | 2.94 x10^-03^ |  |  |  |
| Age category (91 weeks) | -1.40 x10^-02^ | 1.87 x10^-03^ | 1, 89.30 | 56.35 | **<0.001** |
| 1^st^ phase (L) | 1.67 x10^-03^ | 2.54 x10^-03^ | 1, 86.29 | 0.43 | 0.512 |
| 2^nd^ phase (L) | -1.88 x10^-03^ | 2.50 x10^-03^ | 1, 86.47 | 0.56 | 0.454 |
| Sex (M) | 2.88 x10^-03^ | 2.51 x10^-03^ | 1, 86.43 | 1.31 | 0.256 |
| Hatching rank (2^nd^) | 1.81 x10^-03^ | 2.62 x10^-03^ | 1, 86.93 | 0.48 | 0.491 |
| *Age category (91 weeks) x 1^st^ phase (L)* | *2.40 x10^-04^* | *5.28 x10^-03^* | *1, 85.87* | *0.95* | *0.332* |
| *Age category (91 weeks) x 2^nd^ phase (L)* | *-3.58 x10^-03^* | *5.52 x10^-03^* | *1, 85.87* | *0.00* | *0.970* |
| *1^st^ phase (L) x 2^nd^ phase (L)* | *2.85 x10^-03^* | *6.27 x10^-03^* | *1, 85.53* | *1.56* | *0.215* |
| *Age category (91 weeks) x 1^st^ phase (L) x 2^nd^ phase (L)* | *6.88 x10^-03^* | *7.55 x10^-03^* | *1, 85.87* | *0.83* | *0.365* |
| R^2^_m_/ R^2^_c_= 0.20/ 0.43 |  |  |  |  |  |

**Table S4.** Cox proportional hazard models of the relationship between mitochondrial respiration rates (normalised by citrate synthase activity) and flux control ratio (*OxCE*) and survival probability up to April 2022 (i.e., approximately 4 years). Continuous variables were mean centred (mean= 1, SD= 0) to make the coefficients comparable. Note that Exp (coef) are hazard ratios.

|  | Coef ±SE | Exp (Coef) | z | p-value | |
| --- | --- | --- | --- | --- | --- |
| *(a)* **Fixed effects** |  |  |  | |  |
| *ROUTINE* resp. citrate synthase normalised | 0.138 ±0.204 | 1.148 | 0.68 | | 0.500 |
| Sex | -1.551 ±0.514 | 0.211 | -3.02 | | 0.003 |
| **Random effects** | **Variance** |  |  | |  |
| Bird ID | 0.003 |  |  | |  |
| Nest ID | 1.027 |  |  | |  |
|  |  |  |  | |  |
| *(b)* **Fixed effects** |  |  |  | |  |
| *LEAK* resp. citrate synthase normalised | 0.157 ±0.198 | 1.170 | 0.79 | | 0.430 |
| Sex | -1.592 ±0.514 | 0.203 | -3.10 | | 0.002 |
| **Random effects** | **Variance** |  |  | |  |
| Bird ID | 0.003 |  |  | |  |
| Nest ID | 1.046 |  |  | |  |
|  |  |  |  | |  |
| *(c)* **Fixed effects** |  |  |  | |  |
| *OXPHOS* resp. citrate synthase normalised | 0.027 ±0.238 | 1.028 | 0.12 | | 0.910 |
| Sex | -1.496 ±0.508 | 0.224 | -2.94 | | 0.003 |
| **Random effects** | **Variance** |  |  | |  |
| Bird ID | 0.003 |  |  | |  |
| Nest ID | 1.011 |  |  | |  |
|  |  |  |  | |  |
| *(d)* **Fixed effects** |  |  |  | |  |
| *OxCE* | -0.055 ±0.232 | 0.946 | -0.24 | | 0.810 |
| Sex | -1.528 ±0.505 | 0.217 | -3.02 | | 0.002 |
| **Random effects** | **Variance** |  |  | |  |
| Bird ID | 0.003 |  |  | |  |
| Nest ID | 1.013 |  |  | |  |
|  |  |  |  | |  |
| *(e)* **Fixed effects** |  |  |  | |  |
| *Citrate synthase* | -0.134 ±0.239 | 0.874 | -0.56 | | 0.580 |
| Sex | -1.623 ±0.552 | 0.197 | -2.94 | | 0.003 |
| **Random effects** | **Variance** |  |  | |  |
| Bird ID | 0.003 |  |  | |  |
| Nest ID | 1.154 |  |  | |  |
